# Quinoxalinone‐based, endoplasmic reticulum‐targeting photosensitizer nanoparticles for tumor photodynamic therapy

**DOI:** 10.1002/btm2.70170

**Published:** 2026-08-09

**Authors:** Chao Sun, Lici Wang, Tao Li, Lei Duan, Yan Dong, Ji Li

**Affiliations:** ^1^ Department of Pharmacy The Second Qilu Hospital of Shandong University Jinan China; ^2^ Department of Spinal Surgery People's Hospital of Wudi Binzhou China; ^3^ Department of Pharmacy Zibo Central Hospital Zibo China; ^4^ Department of Spinal Surgery The Second Qilu Hospital of Shandong University, Cheeloo College of Medicine, Shandong University Jinan China

**Keywords:** endoplasmic reticulum, nanoparticles, photodynamic therapy, photosensitizer, quinoxalinone

## Abstract

The endoplasmic reticulum (ER) is an indispensable organelle responsible for the synthesis and transport of proteins and membrane lipids, playing a critical role in numerous physiological and pathological processes. Leveraging the properties of the ER, we developed novel quinoxalinone‐based, ER‐targeting photosensitizer nanoparticles (Qui‐PS NPs) by conjugating an ER‐specific targeting peptide(RACR) and evaluated their photodynamic therapy (PDT) efficacy. The size, morphology, cellular uptake, ER targeting capability, cell viability, biodistribution, and antitumor efficacy were assessed using dynamic light scattering, transmission electron microscopy, confocal microscopy, CCK‐8 assay, ICP‐Mass spectrometry, and tumor volume measurements, respectively. The results demonstrated that the synthesized Qui‐PS NPs possessed an average diameter of 79.74±9.4 nm, a polydispersity index (PDI) of 0.23±0.02, and a Zeta potential of −11.63±2.86 mV. These nanoparticles exhibited near‐infrared fluorescence emission centered at 830 nm and demonstrated superior singlet oxygen (^1^O_2_) generation capability. The NPs were readily internalized by MCF‐7 cells, displayed specific ER targeting, and induced cytotoxic effects upon light irradiation, with an $IC_{50}$ value of 3.2±0.06 μg/mL. In tumor‐bearing mice, Qui‐PS NPs preferentially accumulated in tumor tissue and significantly suppressed tumor progression under light irradiation, with minimal impact on body weight. These findings suggest that these ER‐targeted NPs represent a promising nanoplatform for potential application in tumor PDT.

## Graphical abstract

We introduce a novel class of quinoxalinone‐based photosensitizers integrated into a nanoparticle system, specifically engineered for unprecedented targeting efficiency towards the endoplasmic reticulum (ER). This work not only develops a sophisticated biomaterial platform for organelle‐specific PDT but also holds considerable promise for broader applications in targeted drug delivery and combating cancer through modulation of ER function.

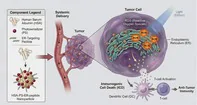

**Translational Impact Statement**

This research addresses the critical need for enhanced precision in cancer photodynamic therapy (PDT). We introduce a novel class of quinoxalinone‐based photosensitizers integrated into a nanoparticle system (Qui‐PS NPs), specifically engineered for unprecedented targeting efficiency towards the endoplasmic reticulum (ER). The strategic selection of the ER as a target aims to leverage its crucial role in cellular stress responses to maximize therapeutic impact. Our study provides compelling evidence, from both in vitro cellular assays and in vivo tumor models, that these ER‐targeted Qui‐PS NPs achieve remarkable tumor growth inhibition under light exposure. We further elucidate the underlying mechanism, confirming the induction of significant ER stress as the primary driver of phototoxicity. The significance of this work lies in the development of a sophisticated biomaterial platform that not only demonstrates substantial efficacy in preclinical models but also pioneers a new approach for organelle‐specific PDT. Furthermore, this platform holds considerable promise for broader applications in targeted drug delivery and could inspire new directions in combating cancer through modulation of ER function.

## 1 INTRODUCTION

Cancer remains a leading cause of mortality worldwide. Conventional therapeutic strategies, including surgery, chemotherapy, and radiation therapy, often exhibit limited efficacy and can significantly impact patients' quality of life. Consequently, there is an urgent need for more effective and novel therapeutic approaches. Among emerging advanced technologies, photodynamic therapy (PDT) represents an increasingly important therapeutic modality, offering spatiotemporal selectivity for treating cancer and other diseases.^1^ The noninvasive nature of PDT allows for minimal damage to surrounding healthy tissues and functions. PDT involves the targeted delivery of photosensitizers (PS) to the tumor microenvironment, followed by irradiation with light of a specific wavelength to initiate photochemical reactions. These reactions generate reactive oxygen species (ROS), which induce cancer cell death via oxidative stress.^2^ In recent years, nanocarrier‐based technologies have been developed to improve the efficient delivery of PS to tumor sites and enhance therapeutic outcomes, potentially synergizing with immunotherapy.^3^

Generally, PDT efficacy can be enhanced by directing PS to critical intracellular organelles such as mitochondria or nuclei.^4^, ^5^ While mitochondrial targeting is common due to its role in initiating anticancer responses, accumulation of PS within mitochondria can sometimes lead to undesired cytotoxicity, particularly in the absence of light.^6^, ^7^ Furthermore, the risk of DNA mutation renders nucleus targeting less desirable. In contrast, the endoplasmic reticulum (ER)—a membrane‐bound organelle responsible for protein synthesis, folding, transport, and maintaining cellular $Ca^{2+}$ homeostasis—is emerging as an ideal subcellular target. ER targeting is gaining significant research interest as it offers a potentially less toxic yet effective strategy for inducing cell death by modulating ROS levels and triggering ER stress pathways.^8^

An effective ER‐targeting PS should ideally possess efficient ^1^O_2_ generation upon excitation with long‐wavelength light, good photostability, and low dark cytotoxicity. However, clinically approved PS, such as porphyrin derivatives, often have rigid, planar, hydrophobic structures that limit their photophysical properties.^9^, ^10^ Therefore, extensive efforts in chemical modification have been undertaken to augment the photostability and ROS generation capabilities of PS.^11^ Recognizing that the quinoxalinone scaffold possesses a high molar absorption coefficient and emits bright blue fluorescence, this chemical structure presents a promising chromophore for designing advanced PS.^12^, ^13^ In this study, we developed novel quinoxalinone‐based, ER‐targeting photosensitizer nanoparticles (Qui‐PS NPs) and investigated their photophysical properties and PDT performance, aiming to provide a promising candidate nanoplatform for tumor PDT.

## 2 RESULTS AND DISCUSSION

### 2.1 Synthesis, chemistry, and photophysical properties of qui‐PS

Developing photosensitizers that absorb light at longer wavelengths is crucial for achieving greater tissue penetration depth, particularly for deep‐seated tumors. To achieve this, the quinoxalinone scaffold, known for its strong blue fluorescence emission, was selected as the core chromophore. By extending the π‐conjugation system through donor units, it is beneficial to increase the maximum absorption wavelength. Through the electron push‐pull effect, it can help reduce the energy barrier between the excited singlet and triplet states, which is conducive to ISC and thereby improves the efficiency of reactive oxygen production.^14^ Accordingly, thiophene and dicyanovinyl groups were conjugated to the quinoxalinone skeleton to achieve high ^1^O_2_ production. The detailed synthetic procedure is illustrated in Scheme 1. As shown in Figure 1, Qui‐PS exhibited broad absorption spanning from 300 to 700 nm. The chemical structure was confirmed by ^1^ *H* NMR and ^13^ *C* NMR spectroscopy, with results presented in Figures S1 and S2, respectively.

**SCHEME 1:**
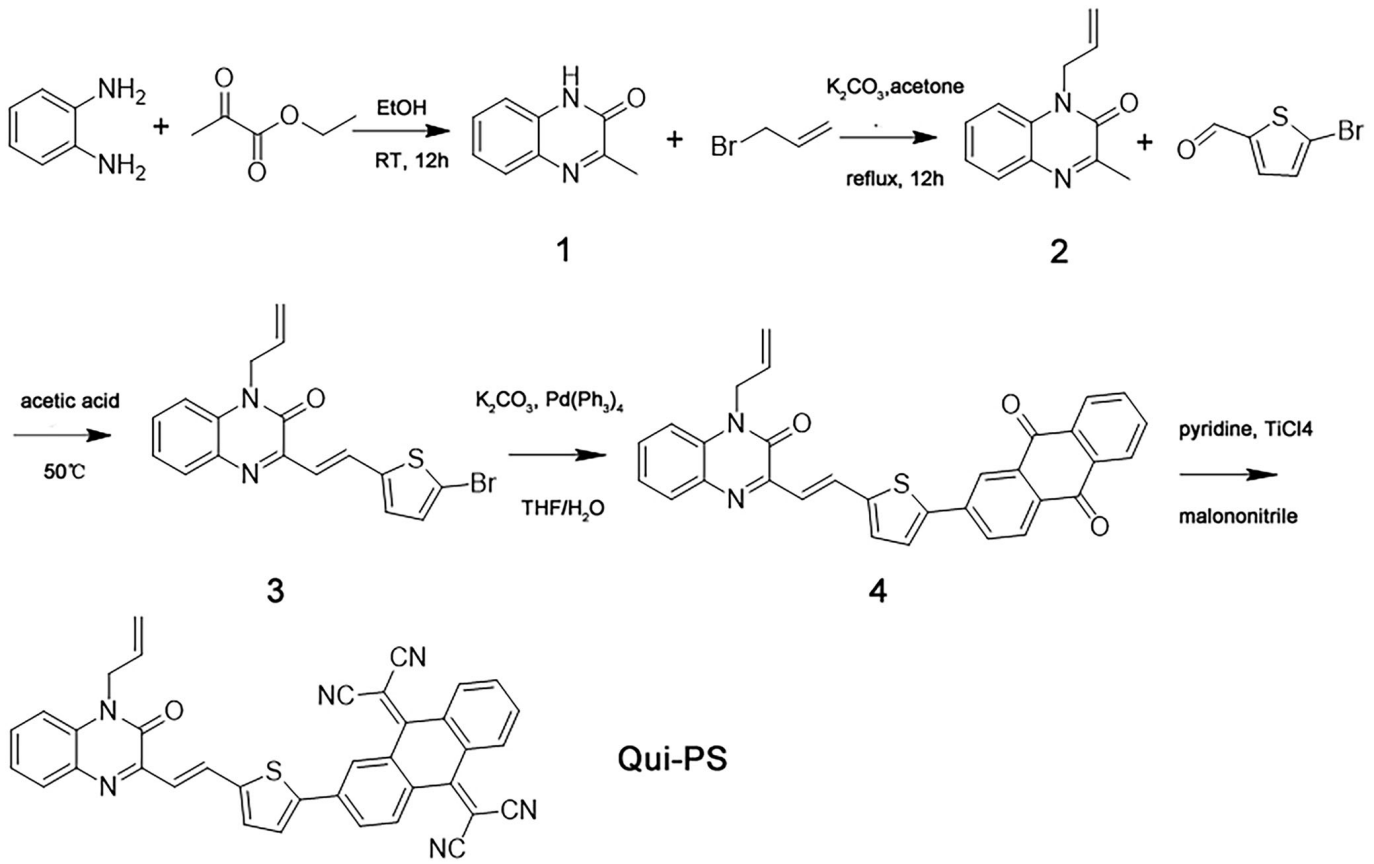
Synthetic procedure of Qui‐PS.

**FIGURE 1:**
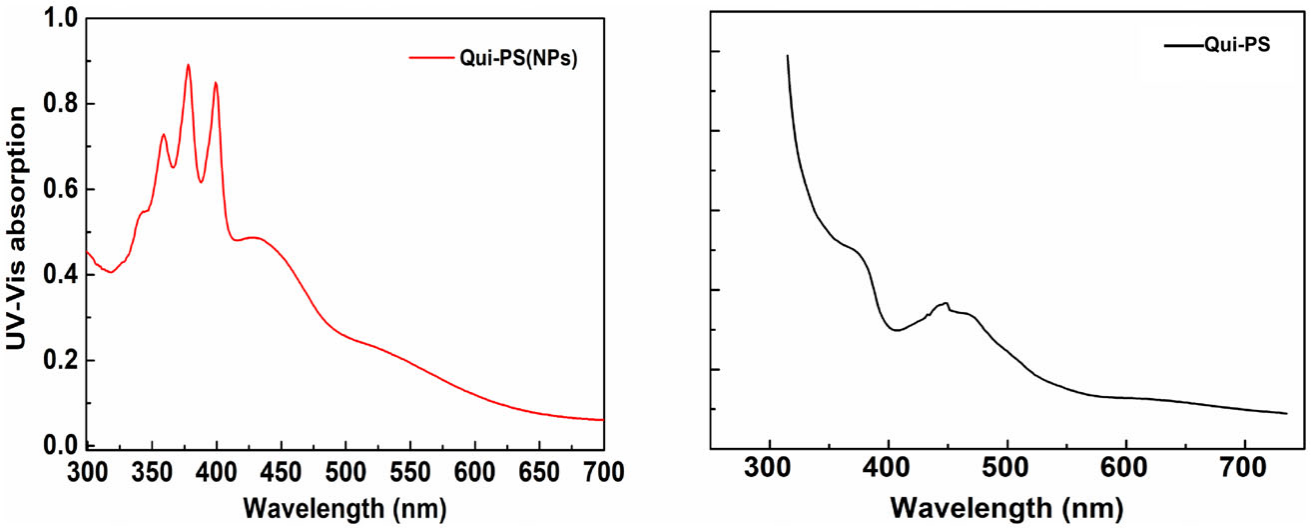
UV–Vis absorption spectra of Qui‐PS(NPs) and Qui‐PS.

### 2.2 Preparation and characterization of qui‐PS NPs

Human serum albumin (HSA), the most abundant protein in human plasma, possesses remarkable binding affinity for various molecules, making it an ideal endogenous carrier for drug delivery, particularly for hydrophobic agents. To improve the aqueous dispersibility and biocompatibility of the hydrophobic Qui‐PS, it was conjugated with HSA to formulate Qui‐PS NPs. And modifying HSA with Reversibly Activated Cell‐Penetrating Peptide (RACR) endow the nanoparticles with tumor‐specific targeting and cell‐penetrating capabilities. RACR can be reversibly activated in the tumor microenvironment (e.g., under specific enzyme expression or pH conditions), thereby enhancing the targeted delivery and intracellular accumulation of Qui‐PS in tumor cells, while reducing off‐target toxicity to normal tissues. The loading efficiency (LE) and encapsulation efficiency (EE) of Qui‐PS in the nanoparticles were (26.7 ± 1.1)% and (89.2 ± 0.8)%, respectively. Dynamic light scattering (DLS) measurements revealed that Qui‐PS NPs had an average hydrodynamic diameter of 79.74±9.4 nm, a low polydispersity index (PDI) of 0.23±0.02, and a negative Zeta potential of −11.63±2.86 mV (Figure 2). Transmission electron microscopy (TEM) images confirmed a uniform, spherical morphology consistent with the DLS data (Figure 2). Due to the conjugation of electron‐donating groups (thiophene and triphenylamine) to the quinoxalinone scaffold, Qui‐PS NPs exhibited NIR fluorescence emission centered at 830 nm (Figure 2c). Also, research of the stability of Qui‐PS nanoparticles in various biological media, including PBS (pH 7.4) and DMEM indicated Qui‐PS nanoparticles had good stability (Figure 3).

**FIGURE 2:**
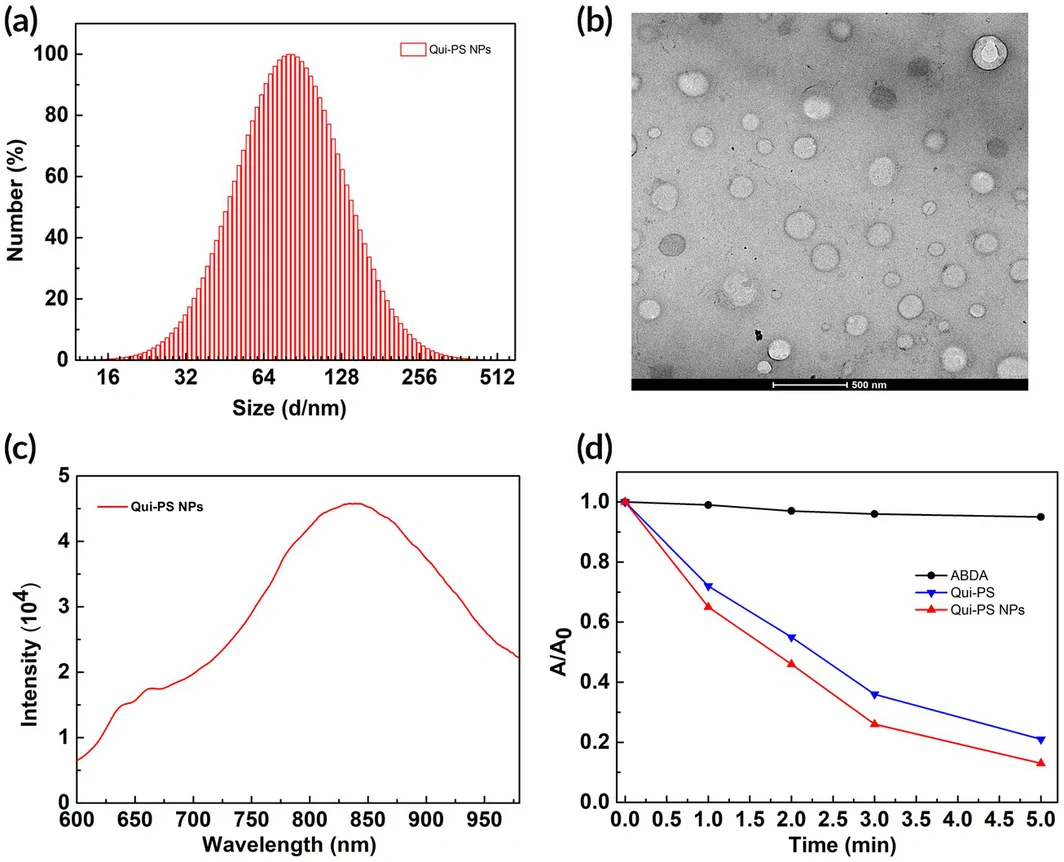
Size distribution, fluorescence intensity, and ^1^O_2_ generation of Qui‐PS NPs. (a) Dynamic light scattering (DLS) study for size distribution of Qui‐PS NPs; (b) Transmission electron microscopy image of Qui‐PS NPs. Bar = 500 nm; (c) Fluorescence emission of Qui‐PS NPs (10 μg/mL, 99% water) with an excitation wavelength of 530 nm; (d) Degradation of ABDA induced by ^1^O_2_.

**FIGURE 3:**
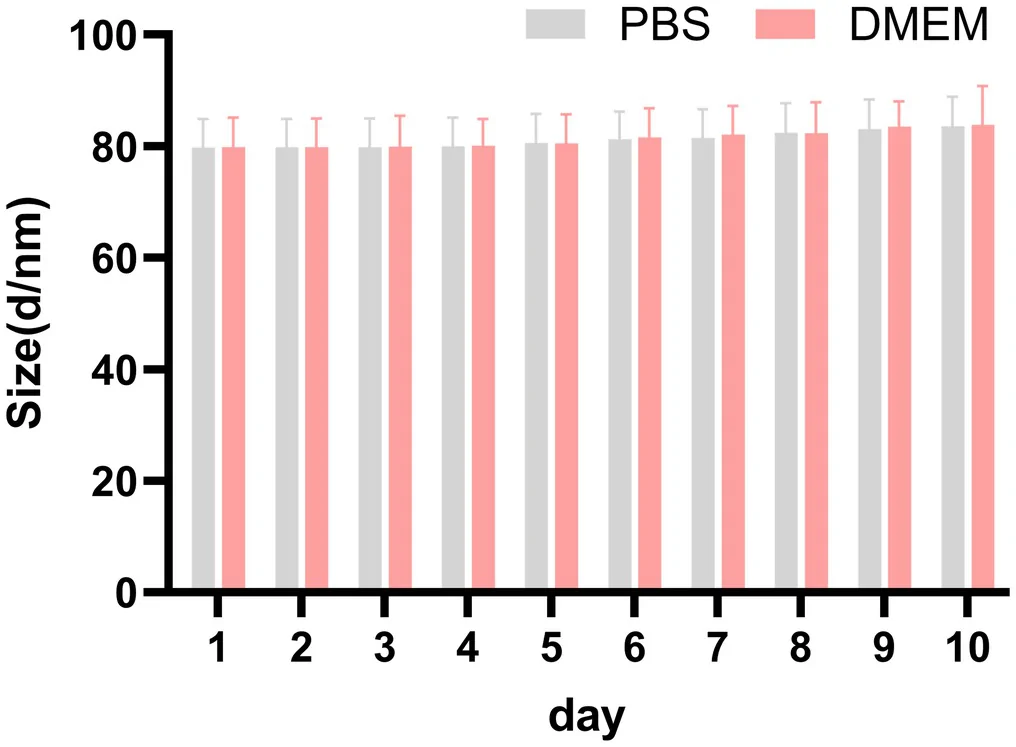
Size distribution of Qui‐PS NPs in PBS and DMEM. Dynamic light scattering (DLS) study for size distribution of Qui‐PS NPs in PBS and DMEM during 10 days.

Rose Bengal (RB) has become a commonly used reference for measuring the singlet oxygen (^1^O_2_) generation quantum yield. The core reasons lie in its high and widely recognized quantum yield, excellent water solubility matching practical application scenarios, stable photophysical properties, as well as low cost and easy availability, making it the gold standard in this field. The ^1^O_2_ generation capability of Qui‐PS NPs was quantified using 9,10‐anthracenediyl‐bis(methylene)dimalonic acid (ABDA) as a probe. As shown in Figure 2d, upon laser irradiation, Qui‐PS NPs caused a rapid decomposition of ABDA, significantly more pronounced than that observed with free Qui‐PS under identical conditions. These results indicate that Qui‐PS NPs possess superior ^1^O_2_ generation efficiency (61.7%), highlighting their potential as effective photosensitizers for PDT.

### 2.3 Cellular uptake, ER targeting, and cell viability study

The cellular uptake, ER targeting ability, and photocytotoxicity of Qui‐PS NPs were subsequently evaluated in MCF‐7 human breast cancer cells. Cells were incubated with Qui‐PS NPs for 2, 4, 6, and 12 h, and uptake was visualized using confocal microscopy. As depicted in Figure 4a, red fluorescence corresponding to the NPs was clearly detected within MCF‐7 cells as early as 2 h post‐incubation. The fluorescence intensity increased progressively over time, indicating efficient time‐dependent cellular internalization of Qui‐PS NPs. Also, quantification of the fluorescence intensity performed by flow cytometry was consistent with the above results (Figure 5).

**FIGURE 4:**
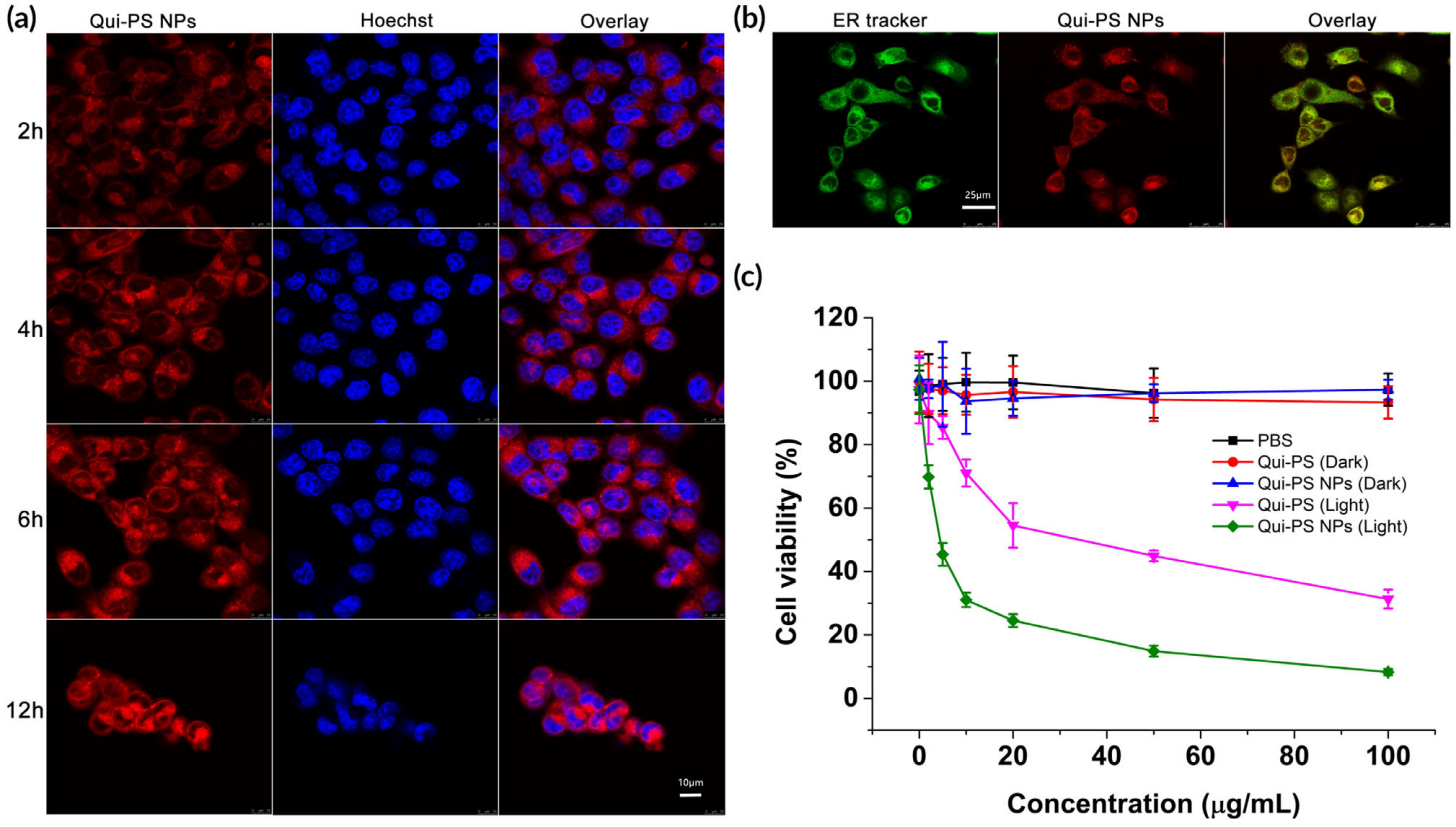
Cell uptake, ER targeting and cell viability of MCF‐7 cells incubated with Qui‐PS NPs (*n* = 3). (a) Confocal fluorescence of MCF‐7 cells after incubation with Qui‐PS NPs (10 μg/mL) for 2, 4, 6 and 12 h, respectively. Bar = 10 μm. (b) Confocal imaging of MCF‐7 cells costained with Qui‐PS NPs (10 μg/mL), ER Tracker, and Hoechst. Cells were incubated with 10 μg/mL for 4 h and ER Tracker for an additional 30 min. Bar = 25 μm; (c) Cell viability study of MCF‐7 cells incubated with PBS, Qui‐PS (Dark), Qui‐PS (Laser), Qui‐PS NPs (Dark), and Qui‐PS NPs (Laser) for 72 h. For light groups, cells were irradiated with 530 nm laser (100 mW/cm^2^) for 5 min.

**FIGURE 5:**
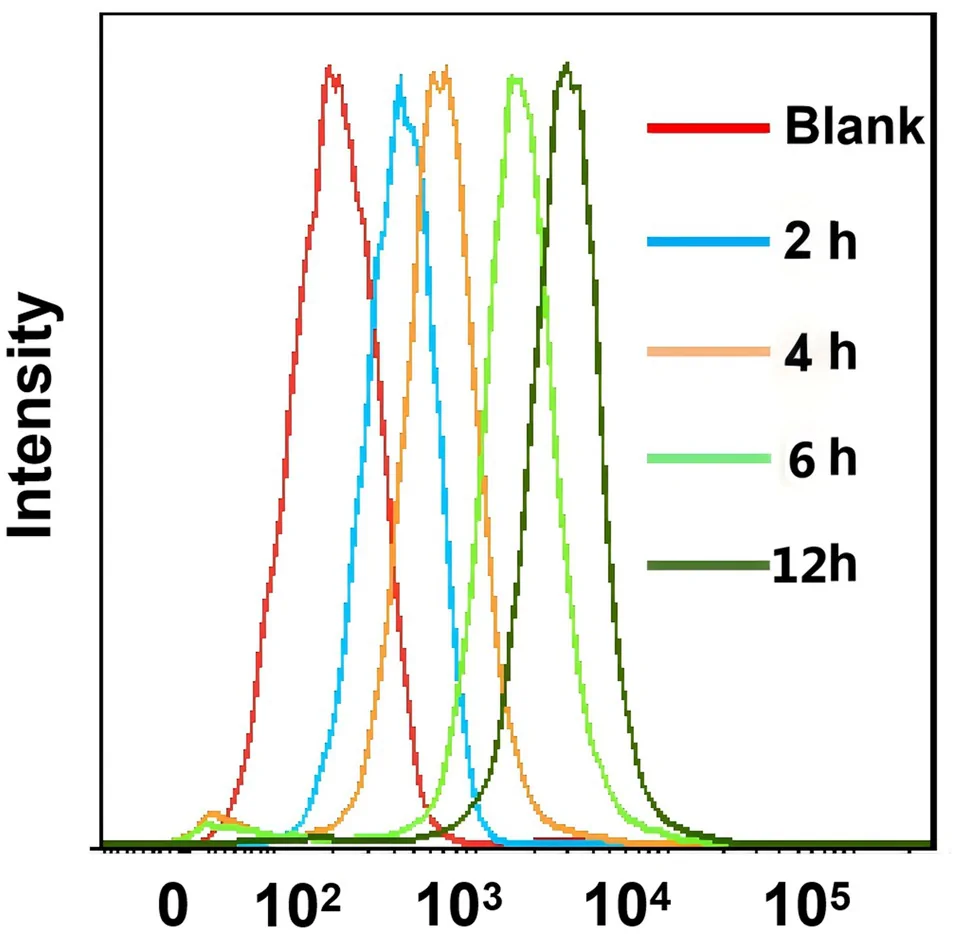
Cell uptake quantification by flow cytometry of MCF‐7 cells incubated with Qui‐PS NPs (*n* = 3). Quantification of the fluorescence intensity performed by flow cytometry of MCF‐7 cells after incubation with Qui‐PS NPs (10 μg/mL) for 2, 4, 6 and 12 h, respectively.

Precise molecular or organelle targeting is a key consideration in the design and development of modern anticancer drugs. Organelle‐targeting nanosystems are regarded as promising strategies for phototherapy, as they can induce localized cytotoxicity with minimal damage to surrounding healthy biological tissues.^15^, ^16^ Arginine‐rich peptides have been reported to facilitate cellular entry and interact with negatively charged drug molecules and organelle membranes via non‐covalent interactions, enabling targeted delivery.^17^, ^18^ In this study, Qui‐PS NPs were functionalized by conjugating an oligo‐peptide (RACR sequence) via a thiol‐ene click reaction to confer ER targeting capability. To confirm this targeting ability, MCF‐7 cells were incubated with Qui‐PS NPs (10 μM) for 4 h, followed by co‐staining with ER‐Tracker Green for 30 min. Confocal microscopy images revealed that the red fluorescence of Qui‐PS NPs showed significant colocalization with the green fluorescence of the ER tracker (Figure 4b), demonstrating effective targeting of the nanoparticles to the ER.

The ER plays vital and multifaceted roles in physiological and pathological processes related to protein and lipid metabolism. ER stress signaling pathways are essential for maintaining ER homeostasis and are considered therapeutic targets for cancer treatment. Given their efficient ^1^O_2_ production and demonstrated ER targeting capability, Qui‐PS NPs were expected to exhibit significant photocytotoxicity towards cancer cells. As shown in Figure 4c, incubation with Qui‐PS NPs followed by light irradiation led to a significant, dose‐dependent decrease in MCF‐7 cell viability. Qui‐PS NPs demonstrated a potent anti‐proliferative effect upon illumination, with an $IC_{50}$ value of 3.2±0.06 μg/mL, which was markedly lower than that of free Qui‐PS under irradiation. Meanwhile, both Qui‐PS NPs and free Qui‐PS exhibited minimal cytotoxicity in the absence of light irradiation, indicating good biocompatibility in the dark.

### 2.4 Antitumor efficacy in tumor‐bearing mice

Encouraged by the favorable photophysical properties and potent in vitro photocytotoxicity, the in vivo antitumor efficacy of Qui‐PS NPs was evaluated in nude mice bearing MCF‐7 tumor xenografts. First, the biodistribution was monitored via in vivo fluorescence imaging after intravenous injection of Qui‐PS NPs (20 mg/kg) via the tail vein. As indicated in Figure 6, the fluorescence signal of Qui‐PS NPs in the tumor region rapidly increased, reaching a peak intensity at 2 h post‐injection, and remained at high levels for up to 12 h. This pattern suggests effective accumulation and retention of Qui‐PS NPs within the tumor tissue, likely facilitated by the enhanced permeability and retention (EPR) effect. Also, ICP‐MS analysis of biodistribution of Qui‐PS in major organs (heart, liver, spleen, lung, kidney, tumor) at different time points post‐injection was performed. As shown in Figure 7, Qui‐PS mainly accumulated in tumor and liver, indicating that it was necessary to pay attention to the impact on the organs during the treatment process, especially the liver.

**FIGURE 6:**
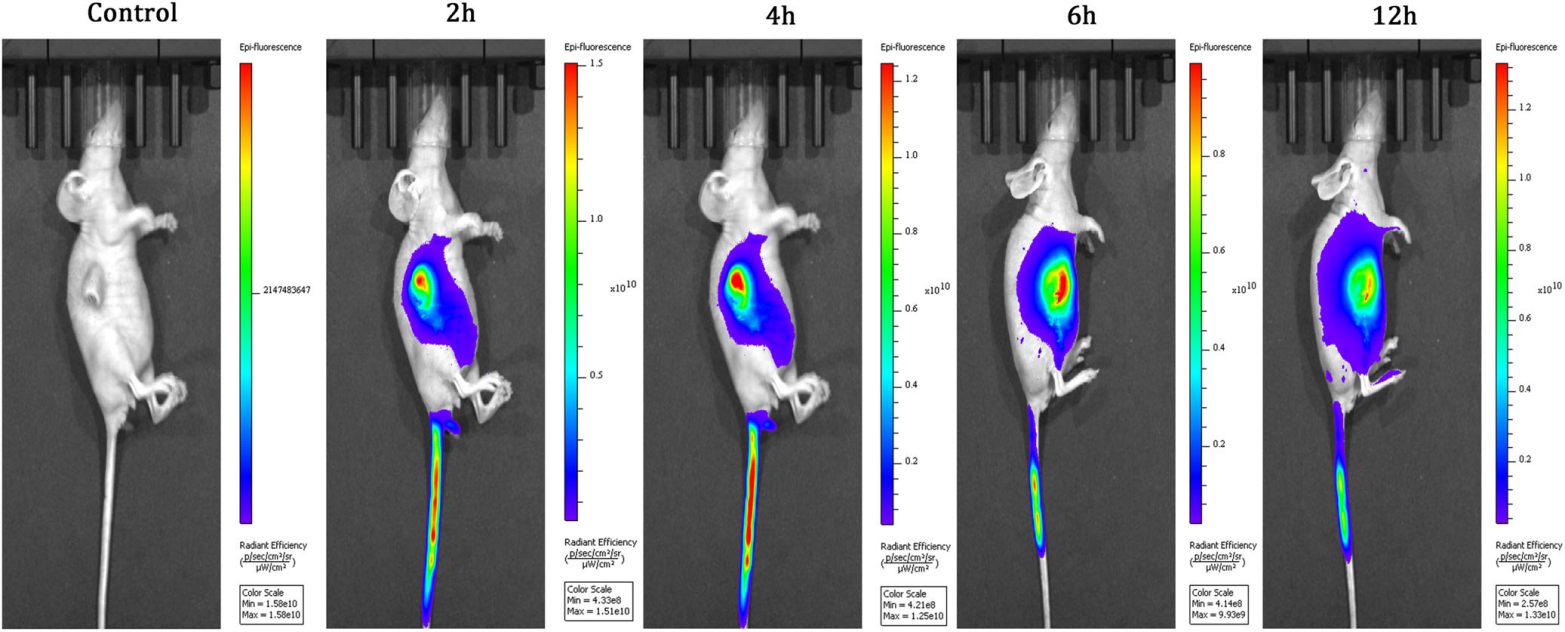
In vivo fluorescent images of tumor‐bearing nude mice after i.v. injection with Qui‐PS NPs for different time (*n* = 5). The MCF‐7 tumor‐bearing nude mice were intravenously injected with Qui‐PS NPs (20 mg/kg) via the tail vein. The accumulation in different tissues at 0, 2, 4, 6, and 12 h after injection was determined by ICP‐Mass, respectively.

**FIGURE 7:**
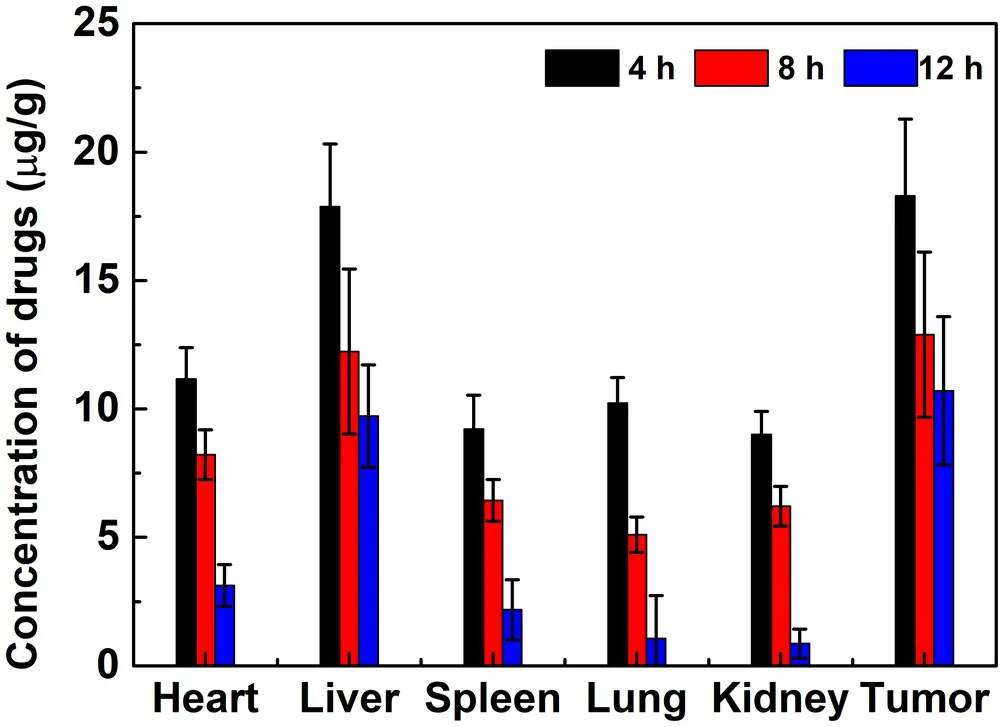
ICP‐MS analysis of biodistribution of Qui‐PS in major organs (heart, liver, spleen, lung, kidney, and tumor) at different time points post‐injection were performed. The MCF‐7 tumor‐bearing nude mice were intravenously injected with Qui‐PS NPs (20 mg/kg) via the tail vein. The accumulation in different tissues at 4, 8, and 12 h after injection was determined by ICP‐Mass, respectively.

The therapeutic efficacy was then assessed. As shown in Figure 8a, tumors in the control group grew progressively, reaching approximately 1400 $mm^{3}$ over 24 days. In contrast, intravenous administration of Qui‐PS NPs followed by laser irradiation almost completely suppressed tumor growth throughout the observation period. This therapeutic effect was significantly superior to that observed with free Qui‐PS under irradiation. Importantly, treatment with Qui‐PS NPs (with or without irradiation) or free Qui‐PS (with irradiation) did not cause any significant changes in mouse body weight, indicating that the treatments were well‐tolerated (Figure 8b). Consistent results were observed in subsequent hematoxylin and eosin (H&E) staining of tumor tissues excised at the end of the experiment (Figure 8c). Tumors from mice treated with Qui‐PS NPs plus laser irradiation showed extensive necrosis and significantly reduced tumor cell density compared to control groups, indicating effective tumor destruction. These preliminary in vivo results underscore the potential of Qui‐PS NPs as an anticancer agent, potentially acting via induction of ER stress upon photoactivation.

**FIGURE 8:**
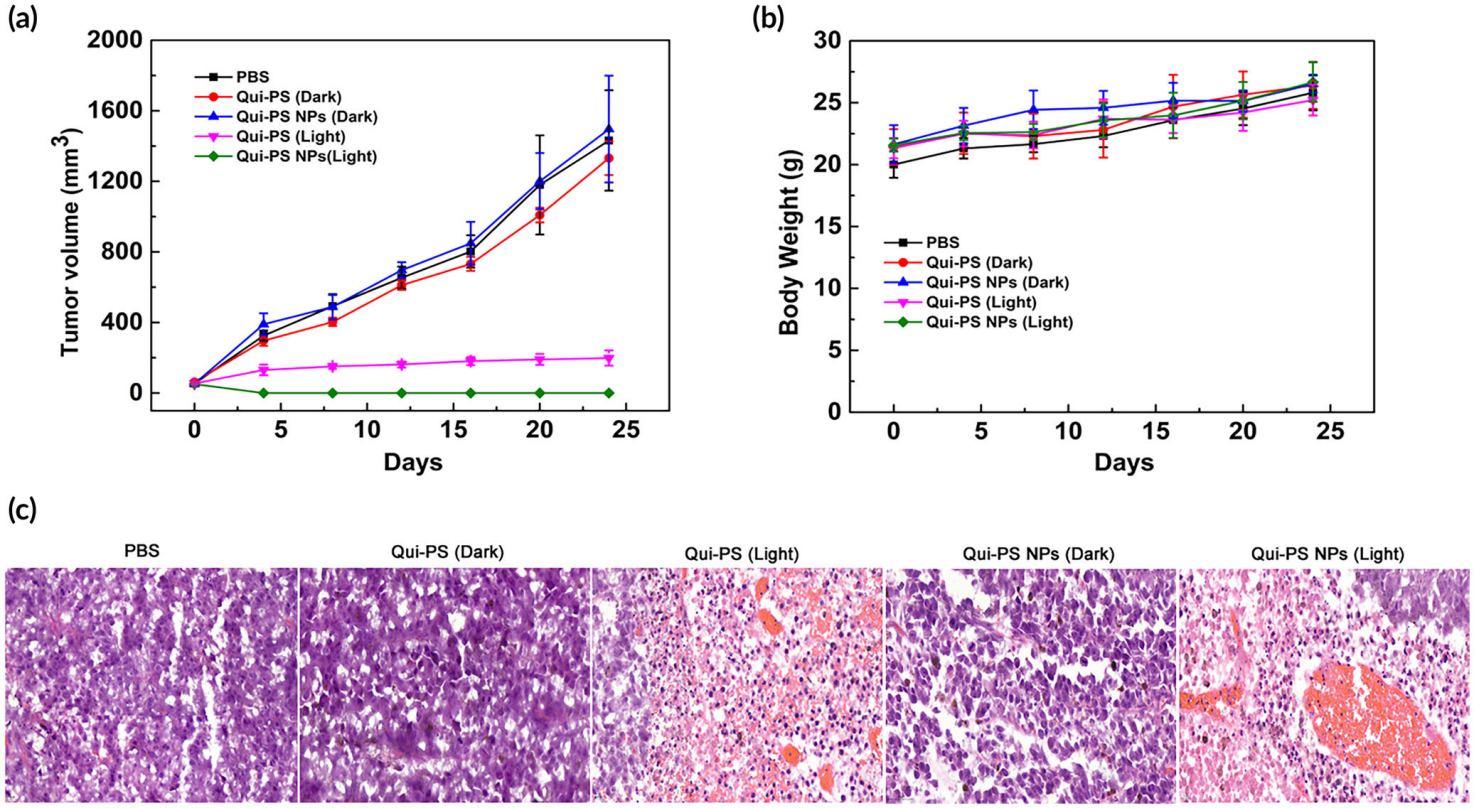
Pharmacodynamics and H&E staining of tumor‐bearing mice after 24 days of treatment (*n* = 5). MCF‐7 tumor‐bearing mice were intravenously injected with PBS, Qui‐PS (20 mg/kg, dark), Qui‐PS (20 mg/kg, light), Qui‐PS NPs (20 mg/kg, dark), and Qui‐PS NPs (20 mg/kg, light) via the tail vein, respectively. For the light groups, mice were treated under a 530 nm laser (100 mW/cm^2^) for 5 min. (a) Tumor volume observed every 4 days during the period of treatment; (b) Body weight observed every 4 days during the period of treatment; (c) H&E staining of tumor tissue sections after treatment. The pink staining indicates the cytoplasm or other components of the tissue and blue staining indicates the nucleus. Original magnification 400×.

Owing to the inadequate infiltration or activation of T lymphocytes within the tumor microenvironment, tumors appeared to be immunogenic cold. Then the tumor infiltrating lymphocytes (TILs) of tumor tissues were investigated to further ascertain whether this molecular‐targeting photodynamic treatment could promote anti‐tumor immunity in the immunogenic cold tumors. As presented in Figure 9, the higher percentages of CD8+ cells (activated cytotoxic T cells) in the tumor tissues of Qui‐PS NPs group (52.7%) and Qui‐PS group (44.1%) were detected in comparison to the tumors of the PBS group (30.1%). Besides, the intratumoral accumulation of CD4+ cells (activated helper T cells) was markedly increased in the Qui‐PS NPs treatment groups (48.1%), whereas only 17.2% of tumor‐infiltrating CD4+ T cells were detected in the PBS group. These results implied that Qui‐PS NPs‐mediated PDT was able to increase tumor infiltrating cytotoxic T cells. We also investigated the cell cycle of tumor tissue cells via flow cytometer. The results showed that Qui‐PS NPs treatment induced a significant G0/G1 phase arrest compared to the control group, indicating that the nanoparticles inhibited cell proliferation by blocking the cell cycle at the G0/G1 phase (Figure 10). Furthermore, we analyzed inflammatory responses caused by the PDT treatment via staining analysis of related cytokines including TNF‐α, IL‐6, IL‐10, IL‐12. Results showed that IL‐10 and IL‐12 were found significantly increased in the tumor tissues, while TNF‐α and IL‐6 significantly decreased (Figure 11).

**FIGURE 9:**
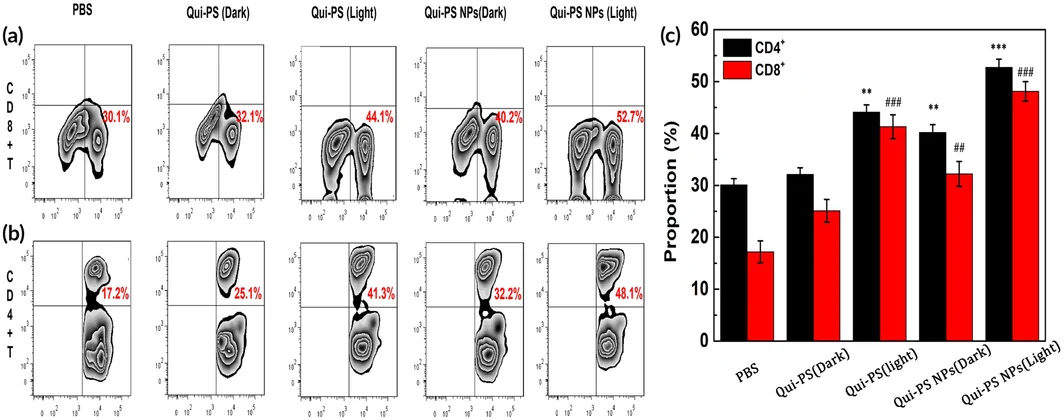
Analysis of T cells a secretion in tumor‐bearing mice (*n* = 5). The female BALB/c mice harboring MCF‐7 tumor model were used to evaluate the influence of CD4+, CD8+ T cells. The tumor‐infiltrating lymphocytes (TILs) were isolated and analyzed via flow cytometry.

**FIGURE 10:**
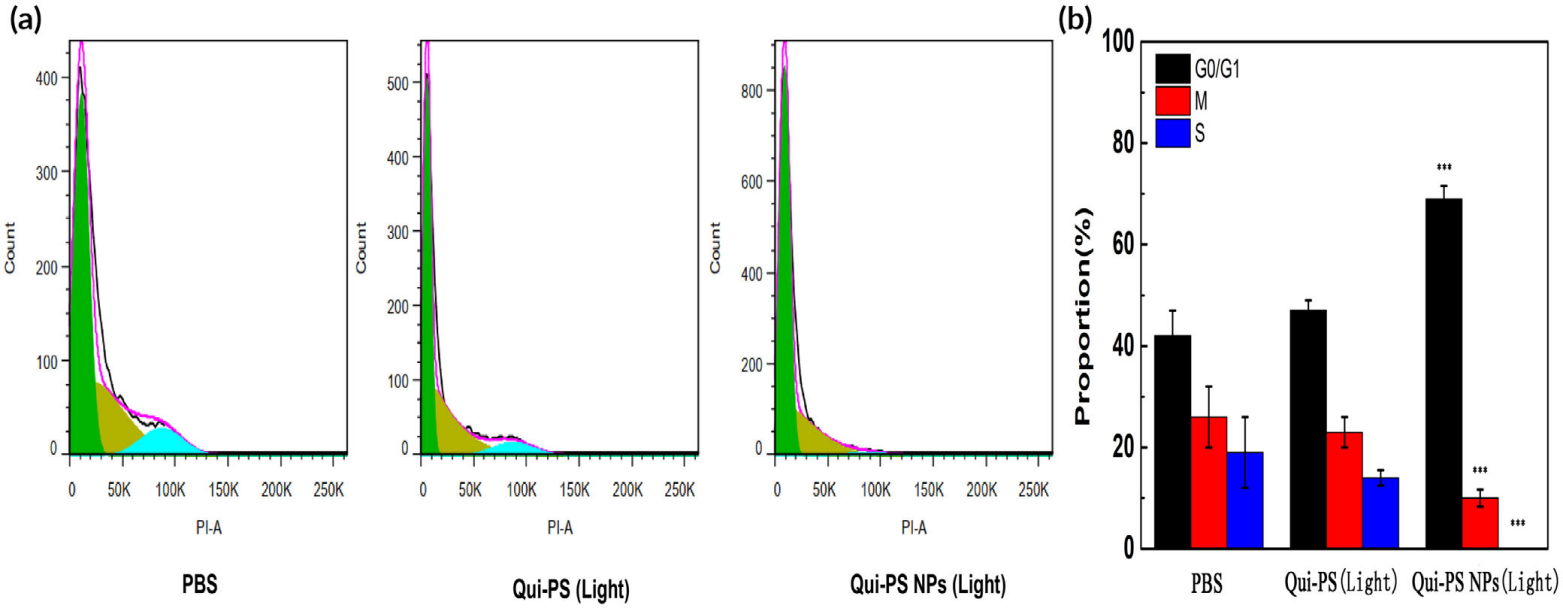
Cell cycle of tumor tissue cells in tumor‐bearing mice (*n* = 5). The female BALB/c mice harboring the MCF‐7 tumor model were used to detect the cell cycle of tumor tissue cells by flow cytometry.

**FIGURE 11:**
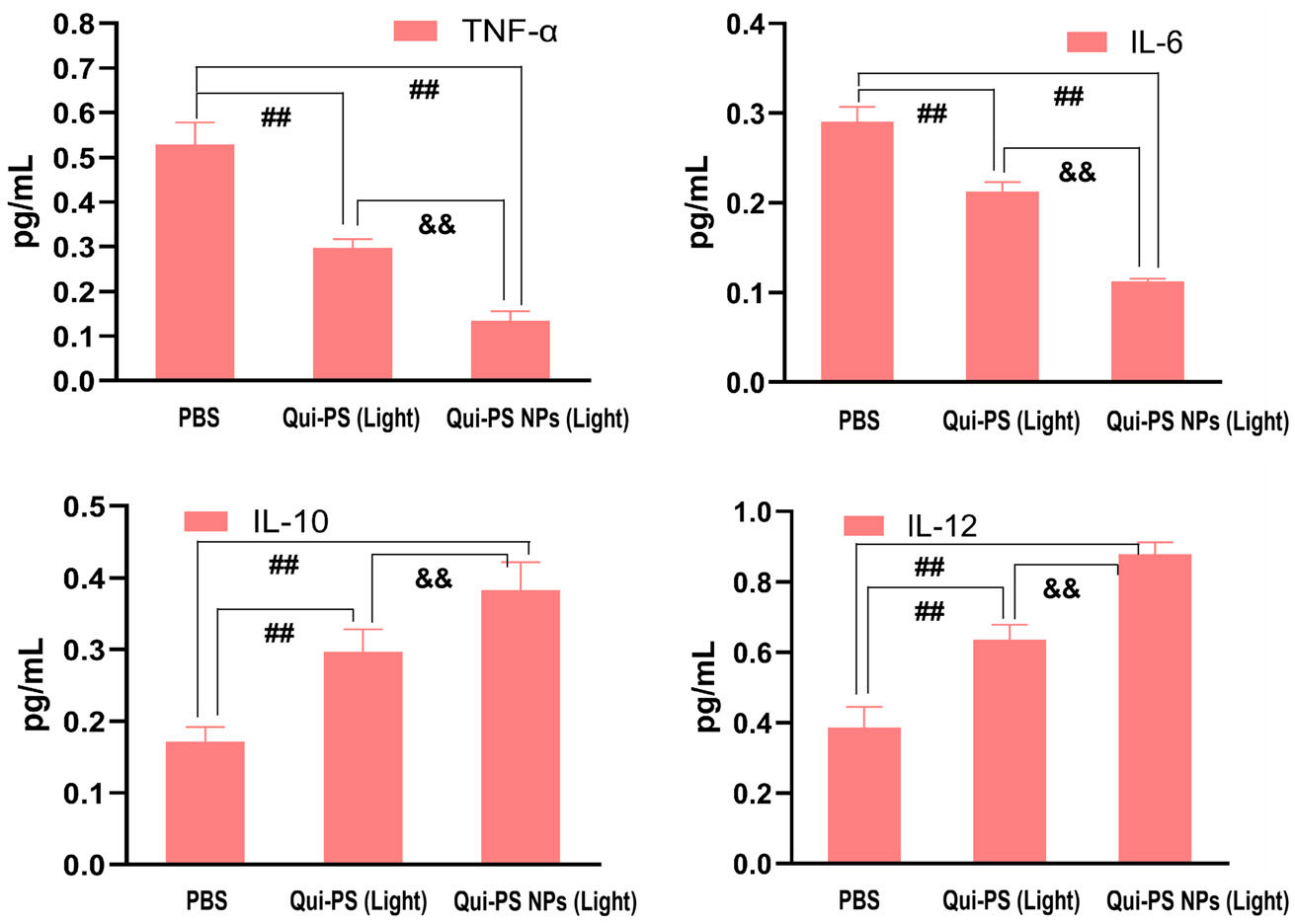
Cytokine secretion in tumor‐bearing mice (*n* = 5). The harvested blood supernatants of female BALB/c mice harboring MCF‐7 tumor mode were collected and cytokines were quantified using mouse TNF‐α, IL‐6, IL‐10, and IL‐12 ELISA kits (Life technologies, USA).

Conventionally, the transformation from cold to hot tumors is considered to depend on the elevation of pro‐inflammatory cytokines. However, excessively high TNF‐α and IL‐6 in tumor tissues contribute to chronic inflammatory microenvironment, facilitate tumor proliferation, invasion, and immune escape, and their moderate reduction after PDT is conducive to alleviating pathological inflammation and weakening pro‐tumor signals.^19^, ^20^ IL‐12 is recognized as a pivotal immunostimulatory cytokine that drives dendritic cell maturation, promotes M1 macrophage polarization, and enhances the infiltration and activation of cytotoxic CD8^+^T cells, which is essential for converting immunologically cold tumors into hot ones.^21^ Moderately increased IL‐10 maintains immune homeostasis, restricts uncontrolled inflammatory responses, and protects normal surrounding tissues from immune injury.^19^ Consistent with previous reports, nano‐PS mediated PDT does not rely on the simple upregulation of pro‐inflammatory cytokines to realize cold tumor transformation.^19^ Instead, it remodels the immune microenvironment by suppressing pathological pro‐inflammatory factors, elevating immunostimulatory IL‐12, and maintaining steady‐state IL‐10 level.^21^ This unique cytokine regulatory profile effectively reverses tumor immune suppression, enhances anti‐tumor immune response, and guarantees the therapeutic efficiency of nanoparticle photosensitization treatment. Collectively, these results demonstrated that the Qui‐PS NPs‐based PDT could potentiate the anti‐tumor immunity response for effective therapy of malignant tumor.

A potential limitation of this study is that the developed PS does not have NIR activation capability. Photosensitizers with NIR emission (650–900 nm) have better tissue penetration depth, which is more conducive to the treatment of deep tumors. The PS used in this study has a fluorescence emission wavelength in the visible region, which may limit its application in deep tumor therapy. In future studies, we will modify the structure of the PS to shift its emission wavelength to the NIR region, thereby improving its tissue penetration ability and clinical application potential.

## 3 CONCLUSIONS

Developing nanoplatforms with precise targeting capabilities represents a promising strategy for advancing tumor therapy. In this study, we successfully constructed ER‐targeted quinoxalinone‐based photosensitizer nanoparticles (Qui‐PS NPs). These nanoparticles demonstrated effective suppression of tumor growth both in vitro and in vivo upon light irradiation, likely mediated by an ER stress‐related mechanism. These preliminary findings indicate that these ER‐targeted PS NPs constitute a potential platform for tumor PDT. We believe this platform could also potentially facilitate the targeted delivery of other therapeutic agents and contribute to opening new avenues for innovative cancer treatments.

## AUTHOR CONTRIBUTIONS

**Lei Duan:** Investigation; formal analysis; writing – review and editing. **Ji Li:** Conceptualization; methodology; data curation; supervision; investigation; writing – review and editing; project administration; funding acquisition. **Tao Li:** Investigation; writing – review and editing; formal analysis. **Chao Sun:** Data curation; investigation; formal analysis; writing – review and editing; writing – original draft; validation; software. **Yan Dong:** Investigation; writing – review and editing; formal analysis. **Lici Wang:** Investigation; validation; formal analysis; data curation; writing – original draft; writing – review and editing; software.

## FUNDING INFORMATION

This work was supported by the Natural Science Foundation of Shandong Province (Grant No. ZR2024MH096).

## CONFLICT OF INTEREST STATEMENT

The authors declare that they have no relevant financial or non‐financial interests to disclose.

## Supporting information


**FIGURE S1.** 1H NMR spectrum of Qui‐PS in chloroform‐*d*.
**FIGURE S2.** ^13^C NMR spectrum of Qui‐PS in chloroform‐*d*.

## Data Availability

The data supporting the findings of this study are available within the article and its Supplementary Information files.
