# Supplementary material for: Quinoxalinone‐based, endoplasmic reticulum‐targeting photosensitizer nanoparticles for tumor photodynamic therapy
Source: Bioeng Transl Med. 2026 Aug 9;11(6):e70170. doi: 10.1002/btm2.70170 (PMC13454413; doi:10.1002/btm2.70170)
Supplement: Supplementary file 1 [file BTM2-11-e70170-s001.docx]

**Quinoxalinone-Based, Endoplasmic Reticulum-Targeting Photosensitizer Nanoparticles for Tumor Photodynamic Therapy**

**Materials and Methods**

1. **Materials**

Benzene-1,2-diamine, ethyl 2-oxopropanoate, and 3-bromoprop-1-ene were purchased from Adamas (China). 5-Bromothiophene-2-carbaldehyde, 5-bromo-[2,2’-bithiophene]-5-carbaldehyde, (4-(diphenylamino)phenyl)boronic acid, and tetrakis(triphenylphosphine)palladium were obtained from J&K Chemical. All reagents were of analytical grade and used without further purification. Fmoc-RACR was purchased from GL Biochem. Endoplasmic Reticulum (ER)-Tracker (endoplasmic reticulum probe), LysoTracker Green (lysosome probe), MitoTracker, and Hoechst (nucleus probe) were purchased from Thermo Fisher. 3-(4,5-Dimethylthiazol-2-yl)-2,5-diphenyltetrazolium bromide (MTT) was acquired from Sigma. Dulbecco’s Modified Eagle Medium (DMEM), Fetal Bovine Serum (FBS), penicillin, and streptomycin were procured from Gibco. All reagents were of analytical grade and used as received.

2. **Instruments**

^1^*H* and ^13^*C* Nuclear Magnetic Resonance (NMR) spectra were recorded on a Mercury Plus 400 MHz spectrometer (Varian, USA) using dimethyl sulfoxide-$d_{6}$ (DMSO-$d_{6}$) and chloroform-$d$ (CDCl_3_) as solvents. Transmission Electron Microscopy (TEM, Tecnai G2 Spirit Biotwin) was employed to examine the morphology and size of the nanoparticles. Dynamic Light Scattering (DLS) measurements were performed using a Malvern Zetasizer Nano S apparatus equipped with a 4.0 mW laser operating at $\lambda$ = 633 nm. Ultraviolet-Visible (UV-Vis) absorption spectra were obtained using a Thermo Electron EV300 UV-Vis spectrophotometer. Steady-state and time-resolved fluorescence measurements were carried out using a PTI-QM/TM/IM steady-state & time-resolved fluorescence spectrofluorometer (USA/CAN) equipped with a Photo Technology International (PTI) LaserStrobe fluorescence system. Absolute fluorescence quantum yields were determined using an integrating sphere.

3. **Synthesis of Qui-PS**

Synthesis of Compound 1: o-Phenylenediamine (0.05 mol, 5.4 g) was dissolved in ethanol (80 mL). A solution of ethyl pyruvate (0.06 mol, 7 g) in EtOH (5 mL) was added dropwise to the mixture over 20 minutes with stirring. The resulting solution was allowed to react at room temperature overnight. The precipitate formed was collected by filtration, washed with ethanol, and dried under vacuum to yield compound 1 (6.7 g, 84% yield) as a white solid.

Synthesis of Compound 2: Compound 1 (10 mmol, 1.6 g) and K_2_CO_3_ (12 mmol, 1.65 g) were suspended in acetone (50 mL). A solution of 3-bromoprop-1-ene (12 mmol, 1.44 g) was added, and the reaction mixture was heated under reflux in an oil bath for 8 hours. Acetone was subsequently removed using a rotary evaporator. The residue was resuspended in distilled water (10 mL) and ethyl acetate (EA) (20 mL). The organic layer was separated, dried over anhydrous MgSO_4_, filtered, and concentrated via rotary evaporation to obtain the crude product. Further purification by silica gel column chromatography (hexane:EA = 25:1, v:v) afforded compound 2 (1.1 g, 54% yield) as a white crystalline solid. ${}^{1}$H NMR (400 MHz, CDCl_3_, $\delta$): 7.83 (d, $J=8.0$ Hz, 1H), 7.49-7.51 (m, 1H), 7.33-7.35 (m, 1H), 7.28 (d, $J=8.0$ Hz, 1H), 5.92-5.98 (m, 1H), 5.28 (d, $J=8.0$ Hz, 1H), 5.18 (d, $J=8.0$ Hz, 1H), 4.90 (d, $J=8.0$ Hz, 2H), 2.61 (s, 3H) ppm. HRMS (ESI): $m/z$ calculated for C_12_H_12_N_2_O [M]^+^, 201.0950; found 201.0984.

Synthesis of Compound 3: To a mixture of 5-bromothiophene-2-carbaldehyde (3 mmol, 570 mg) and 2-bromoanthracene-9,10-dione (0.5 mmol, 187 mg) were added K_2_CO_3_ (2 mmol, 2.76 g), 2-(4,4,5,5-tetramethyl-1,3,2-dioxaborolan-2-yl)anthracene-9,10-dione (0.6 mmol, 201 mg), tetrakis(triphenylphosphine)palladium (0.05 mmol, 57 mg), distilled water (10 mL), and 1,4-dioxane (20 mL). The reaction mixture was stirred at 80 °C under an argon atmosphere for 72 hours. The mixture was then concentrated using a rotary evaporator and partitioned between water (20 mL) and ethyl acetate (50 mL). The organic layer was collected, dried over anhydrous MgSO_4_, and purified by silica gel column chromatography (PE:EA = from 20:1 to 10:1) to yield compound 3 (522 mg, 55% yield) as a yellow solid. ${}^{1}$H NMR (400 MHz, CDCl_3_, $\delta$): 8.08-8.12 (m, 1H), 7.78 (d, $J=8.0$ Hz, 1H), 7.52-7.57 (m, 2H), 7.42-7.45 (m, 1H), 7.31-7.37 (m, 1H), 7.24-7.27 (m, 2H), 5.87-5.98 (m, 1H), 5.18 (d, $J=8.0$ Hz, 1H), 5.09 (d, $J=8.0$ Hz, 1H), 4.86-4.90 (m, 2H) ppm. HRMS (ESI): $m/z$ calculated for C_17_H_13_BrN_2_OS [M]^+^, 373.2669; found 373.0002.

Synthesis of Compound 4: Compound 2 (0.5 mmol, 100 mg), compound 3 (0.5 mmol, 159 mg), and a catalytic amount of concentrated sulfuric acid were combined. The resulting solution was heated to 50 °C and reacted for 12 hours. The organic layer was collected, dried over anhydrous MgSO_4_, and purified by silica gel chromatography (PE:EA = from 100:1 to 20:1) to yield compound 4 (105 mg, 42% yield) as a red solid. ${}^{1}$H NMR (400 MHz, CDCl_3_, $\delta$): 8.54-8.57 (m, 1H), 8.42 (d, $J=8.0$ Hz, 1H), 8.34-8.38 (m, 3H), 8.03 (dd, $J_{1}=8.0$ Hz, $J_{2}=2.0$ Hz, 1 H), 7.97 (d, $J=8.0$ Hz, 1H), 7.82-7.86 (m, 2H), 7.65 (d, $J=8.0$ Hz, 1H), 7.58-7.60 (m, 1H), 7.51-7.54 (m, 1H), 7.39-7.43 (m, 2H), 7.33 (d, $J=8.0$ Hz, 1H), 5.95-6.04 (m, 1H), 5.33 (d, $J=8.0$ Hz, 1H), 5.24 (d, $J=8.0$ Hz, 1H), 4.98-5.00 (m, 2H). HRMS (ESI): $m/z$ calculated for C_31_H_20_BrN_2_O_3_S [M]^+^, 501.5720; found 501.4521.

Synthesis of Qui-PS: Compound 4 (0.2 mmol, 100 mg) and malononitrile (0.3 mmol, 20 mg) were dissolved in dichloromethane (10 mL). Titanium tetrachloride (0.4 mmol, 40 $\mu$L) was slowly added at 0 °C. After stirring for 30 minutes, pyridine (0.4 mmol, 40 $\mu$L) was injected, and stirring was continued for another 30 minutes. The mixture was then heated at 45 °C for 48 hours. After cooling to room temperature, the reaction was quenched with water (30 mL), and the mixture was extracted with dichloromethane. The combined organic layer was washed with brine, dried over Na_2_SO_4_, and concentrated under reduced pressure. The residue was purified by column chromatography using n-hexane/dichloromethane (1/2, v/v) to yield the desired product, Qui-PS, as a black powder. Qui-PS was subsequently redissolved in CH_3_CN and purified by High-Performance Liquid Chromatography (HPLC) (CH_3_CN:H_2_O = from 40:60 to 95:5 with 0.1% trifluoroacetic acid (TFA)) to afford Qui-PS (51 mg, 27% yield) as a black powder. The purity of Qui-PS was confirmed by HPLC analysis based on peak area ratio, indicating nearly 100% purity. ${}^{1}$H NMR (400 MHz, CDCl_3_, $\delta$): 8.49-8.51 (m, 1H), 8.38-8.41 (m, 1H), 8.27-8.33 (m, 3H), 7.93-7.98 (m, 2H), 7.76-7.79 (m, 2H), 7.61-7.65 (m, 1H), 7.48-7.53 (m, 2H), 7.34-7.38 (m, 2H), 7.29 (d, $J=8.0$ Hz, 1H), 5.92-6.03 (m, 1H), 5.31 (d, $J=8.0$ Hz, 1H), 5.20 (d, $J=8.0$ Hz, 1H), 4.98-5.00 (m, 2H). ${}^{13}$C NMR (100 MHz, CDCl_3_, $\delta$): 159.9, 159.4, 154.5, 150.8, 145.3, 141.7, 137.9, 132.6, 131.1, 130.2, 130.0, 129.8, 128.2, 127.6, 127.4, 124.4, 118.1, 113.7, 112.2, 108.6, 44.3 pp. HRMS (ESI): $m/z$ calculated for C_37_H_20_BrN_6_OS [M]^+^, 597.6680; found 597.1467.

4. **Preparation and Characterization of Qui-PS NPs**

Synthesis of RACR-HSA: Initially, 2 mL of PBS containing 10 mg of HSA was mixed with 0.73 mg of sulfo-SMCC. After stirring for 2 hours at room temperature, excess sulfo-SMCC was removed using a centrifugal filter device (MWCO = 10.0 kDa) to purify the SMCC-activated HSA. SMCC-activated HSA and Reversibly Activated Cell-Penetrating Peptide (RACR) were combined in PBS (pH 7.4) at a molar ratio of 1:10. Following a 24-hour reaction at 4 °C, excess RACR was removed using the centrifugal filter device (MWCO = 10.0 kDa).

Preparation of Qui-PS NPs: Qui-PS and RACR-HSA were added to 2 mL of ultra-pure water. The solution was then sonicated for 30 minutes and stirred overnight at room temperature. Any resulting precipitation was removed by high-speed centrifugation. The obtained nanoparticles were stored in a dark environment. The loading efficiency (LE) and encapsulation efficiency (EE) of Qui-PS in the nanoparticles were measured by high-performance liquid chromatography (HPLC). For cellular studies, the stock solution was further diluted with PBS to a concentration of 20 $\mu$M (all concentration values refer to the concentration of Qui-PS encapsulated in the nanoparticles). The stability of Qui-PS NPs was assessed by monitoring size variations using DLS at different time points.

5. **^1^O_2_  Quantum Yield Determination**

9,10-Anthracenediyl-bis(methylene)dimalonic acid (ABDA) was utilized as the ^1^O_2_ indicator. 10 $\mu$L of ABDA solution (2 mol/L) was added to 1 mL of either Qui-PS or Qui-PS NPs solution. The mixtures were then irradiated with white light (400-800 nm, power density 0.25 W/cm^2^) for varying durations. The absorbance of ABDA at 378 nm was measured to determine the decay rate of the photosensitizing process, using Rose Bengal (RB) as the standard photosensitizer. The ^1^O_2_ quantum yield in water ($\Phi_{PS}$) was calculated using the formula presented as following:

where $K_{PS}$ and $K_{RB}$ represent the decomposition rate constants of ABDA induced by the photosensitizer (PS) and RB, respectively, and $A_{PS}$ and $A_{RB}$ represent the integrated absorbance of PS and RB in the 400-800 nm range. The ^1^O_2_ quantum yield of RB ($\Phi_{RB}$) in water is 0.75.

6. **Cell Culture**

MCF-7 cells were maintained in Dulbecco’s Modified Eagle Medium (DMEM) supplemented with 10% Fetal Bovine Serum (FBS) and antibiotics (50 units/mL penicillin and streptomycin) at 37°C in a humidified atmosphere containing 5% CO_2_.

7. **Cellular Uptake Study of Qui-PS NPs in MCF-7 Cells**

MCF-7 cells were seeded in 6-well plates and cultured overnight. The cells were then incubated with Qui-PS NPs under static conditions for 2, 4, and 12 hours, respectively. Subsequently, cells were fixed with 4% formaldehyde and stained with Hoechst (5 $\mu$g/mL). Cellular uptake efficiency was visualized using confocal microscopy and quantified by flow cytometry. Hoechst fluorescence was excited at 405 nm, with emission collected between 430-490 nm. Qui-PS NPs fluorescence was excited at 488 nm, with emission collected between 600-700 nm.

8. **In Vitro Cell Viability Studies**

MCF-7 cells were seeded in 96-well plates and cultured overnight. Cells were then treated with various concentrations of Qui-PS NPs and incubated for 72 hours. Following incubation, 20 $\mu$L of MTT solution (5 mg/mL) was added to each well. After a 4-hour incubation at 37°C, the medium was replaced with 200 $\mu$L of DMSO. The absorbance of the resulting solution was measured at 570 nm.

9. **Animals and Tumor Models**

Female Balb/c nude mice (5 weeks old) were obtained from the Center of Experimental Animals, Shandong University (Jinan, China). All animal experiments were conducted in accordance with the guidelines of The National Regulation of China for Care and Use of Laboratory Animals. Nude mice were subcutaneously injected with 200 $\mu$L of a cell suspension containing 5 x 10^6^ MCF-7 cells. Tumors were allowed to grow to approximately 200 mm^3^ before initiating experiments.

10. **In Vivo Imaging Study**

MCF-7 tumor-bearing nude mice were intravenously injected via the tail vein with 200 $\mu$L of Qui-PS NPs (20 mg/kg). Mice without any treatment served as the negative control group (n=5). Fluorescence intensity was monitored at 2, 4, 6, and 12 hours post-injection using an in vivo imaging system with appropriate excitation and emission wavelengths ($\lambda_{ex}$ = 488 nm, $\lambda_{em}$ = 652 nm).

11. **In Vivo Pharmacodynamics**

MCF-7 tumor-bearing nude female mice were randomly divided into 5 groups (n=5). The groups received intravenous injections via the tail vein of PBS, Qui-PS (20 mg/kg, dark), Qui-PS (20 mg/kg, light), Qui-PS NPs (20 mg/kg, dark), or Qui-PS NPs (20 mg/kg, light), respectively. For the ‘light’ groups, mice were irradiated with a 530 nm laser (100 mW/cm^2^) for 5 minutes post-injection. Tumor volume and body weight were recorded every four days over a 24-day period following the first injection. Tumor volume was calculated using the formula: $V\left( mm^{3} \right)=1/2\times\text{width}\left( mm \right)^{2}\times\text{length}\left( mm \right)$.

**12. The analysis of T cells, cell cycle and cytokine secretion in vivo**

After 24-day various treatments, five mice per group were sacrificed and the tumors, spleens, and blood were harvested for detection. The tissues were digested with collagenase IV (175 U/mL), hyaluronidase (100 U/mL), and DNase (30 U/mL) at 37 °C for 60 min. The tumor-infiltrating lymphocytes (TILs) were isolated by passing 75 μm filters and enriched for the following analysis. For the T cells frequency detection, the TILs were labeled with anti-CD3-APC, anti-CD4-FITC, and anti-CD8-PE. The stained TILs were then washed thrice with PBS and analyzed via flow cytometry.

Tumor tissues were stripped and cut into small pieces. The tissue fragments were digested with 0.25% trypsin at 37 °C for 30 min to prepare single-cell suspensions. The cell suspension was then filtered through a 75 μm cell strainer to remove tissue debris and washed twice with pre-cooled PBS. Subsequently, the cells were fixed overnight with 70% ice-cold ethanol at 4 °C. After fixation, the cells were washed with PBS to remove ethanol, and stained with propidium iodide (PI) staining solution containing RNase A at 37 °C for 30 min in the dark. Finally, the cell cycle distribution was detected and analyzed by flow cytometry.

For cytokine quantification, the harvested spleen cells were suspended in 5 mL of the DMEM culture medium supplemented with 10 % FBS for 24 h. The supernatants were collected and cytokines were quantified using mouse IL-4, IL-6, IL-12, and IFN-γ ELISA kits (Life technologies, USA).

**13. Statistical Analysis**

Results are presented as mean ± standard deviation (SD). Differences between two groups were assessed using the Student’s t-test via SPSS software. A p-value less than 0.05 (p < 0.05) was considered statistically significant. ‘NS’ indicates no significant difference.

**Figure S1**. 1H NMR spectrum of Qui-PS in chloroform-*d*

**Figure S2**. ^13^C NMR spectrum of Qui-PS in chloroform-*d*
